# Deep learning enables automated volumetric assessments of cardiac function in zebrafish

**DOI:** 10.1242/dmm.040188

**Published:** 2019-10-01

**Authors:** Alexander A. Akerberg, Caroline E. Burns, C. Geoffrey Burns, Christopher Nguyen

**Affiliations:** 1 Cardiovascular Research Center, Massachusetts General Hospital, Charlestown, MA 02129, USA; 2 Harvard Medical School, Boston, MA 02115, USA; 3 Department of Cardiology, Boston Children's Hospital, Boston, MA 02115, USA; 4 Harvard Stem Cell Institute, Cambridge, MA 02138, USA; 5 Athinoula A Martinos Center for Biomedical Imaging, Charlestown, MA 02129, USA

**Keywords:** Zebrafish embryos, CFIN, Ejection fraction, Light-sheet fluorescence microscopy, LSFM

## Abstract

Although the zebrafish embryo is a powerful animal model of human heart failure, the methods routinely employed to monitor cardiac function produce rough approximations that are susceptible to bias and inaccuracies. We developed and validated a deep learning-based image-analysis platform for automated extraction of volumetric parameters of cardiac function from dynamic light-sheet fluorescence microscopy (LSFM) images of embryonic zebrafish hearts. This platform, the Cardiac Functional Imaging Network (CFIN), automatically delivers rapid and accurate assessments of cardiac performance with greater sensitivity than current approaches.

This article has an associated First Person interview with the first author of the paper.

## Abstract (precis)

**Summary:** The authors present CFIN, a deep learning-based image-analysis platform to automatically analyze dynamic light-sheet fluorescence microscopy images and determine volumetric indices of cardiac function in embryonic zebrafish.

## INTRODUCTION

Despite the advantages of utilizing zebrafish embryos for modeling human heart failure (Bakkers, 2011; Becker et al., 2012; Chen et al., 1996; Liu et al., 2014; Shih et al., 2015; Stainier et al., 1996), the metrics employed to monitor cardiac function remain limited and unrefined. The most common metric, ‘fractional shortening’ (FS), is a proxy for volumetric output calculated from the cross-sectional diameters of the ventricle during diastole (chamber relaxation) and systole (chamber contraction), which are measured from two-dimensional (2D) images (Hoage et al., 2012; Yalcin et al., 2017). Despite its ease of use, FS suffers from inconsistencies caused by variable imaging angles, and subjective identification of systole, diastole and cross-sectional diameter. Furthermore, the accuracy of this method relies upon the assumption that chamber diameter is uniformly correlated with chamber volume, which is not always the case. Regional abnormalities in wall movement can over- or underestimate functional deficits depending on where the abnormalities reside relative to the diameters being measured. In short, the most widely used indicator of cardiac performance in zebrafish embryos relies on indirect, non-volumetric measurements that are subject to confounding factors and inaccuracies.

By contrast, functional indices derived from direct measurements of chamber volume, such as ejection fraction (EF) and cardiac output (CO), do not suffer from the same weaknesses. The imaging modalities required to obtain these measurements in larger subjects, such as three-dimensional (3D) echocardiography and magnetic resonance imaging (MRI), are not adaptable to the small scale of zebrafish embryos. Recently, four-dimensional (4D) reconstructions of the embryonic zebrafish heart from dynamic light-sheet fluorescence microscopy (LSFM) images have been shown to offer unparalleled spatial and temporal resolution with which to characterize cardiac morphology and chamber wall dynamics (Lee et al., 2016; Mickoleit et al., 2014). However, quantifying dynamic chamber volumes from these datasets, especially from multiple subjects, remains impractical because it requires the tedious manual segmentation of atrial and ventricular chambers in thousands of component static images.

While various traditional computational approaches exist to automate image segmentation (Sharma and Aggarwal, 2010), many of these methods still require some level of human input and can be limited in their ability to segment and classify distinct, yet similar, structures within biomedical images (Fei et al., 2016; Krämer et al., 2009; Packard et al., 2017). Several of these algorithms, including watershed segmentation, were previously evaluated for their ability to segment fluorescent images of embryonic zebrafish hearts at 48 hours post-fertilization (hpf) (Krämer et al., 2009). While these methods were capable of segmenting whole hearts from background, they failed to consistently delineate individual cardiac chambers. Even if this shortcoming could be overcome, these approaches would only incrementally reduce user workload because the manual annotation of ‘atrium’ and ‘ventricle’ would still be required for each image to calculate chamber volumes.

Convolutional neural networks (CNNs), a class of deep learning neural network, can overcome many of these limitations by applying unsupervised, higher-level and adaptable segmentation criteria to image analysis, while concurrently increasing speed and reducing the amount of processing power required (Litjens et al., 2017). When compared to other approaches, CNNs have been shown to best match the performance of human experts when analyzing biomedical images from numerous sources, including computed tomography (CT), MRI and fluorescence microscopy (Bai et al., 2018; Ciompi et al., 2015; Dou et al., 2017; Hay and Parthasarathy, 2018; Zhang et al., 2019). Therefore, we utilized a deep learning CNN to implement a novel image-analysis platform we termed Cardiac Functional Imaging Network (CFIN). CFIN is designed to: (1) automatically and rapidly segment atrial and ventricular boundaries within LSFM datasets of live embryonic zebrafish hearts, and (2) utilize these data to calculate volumetric indices of cardiac function.

## RESULTS

**Fig. 1.**
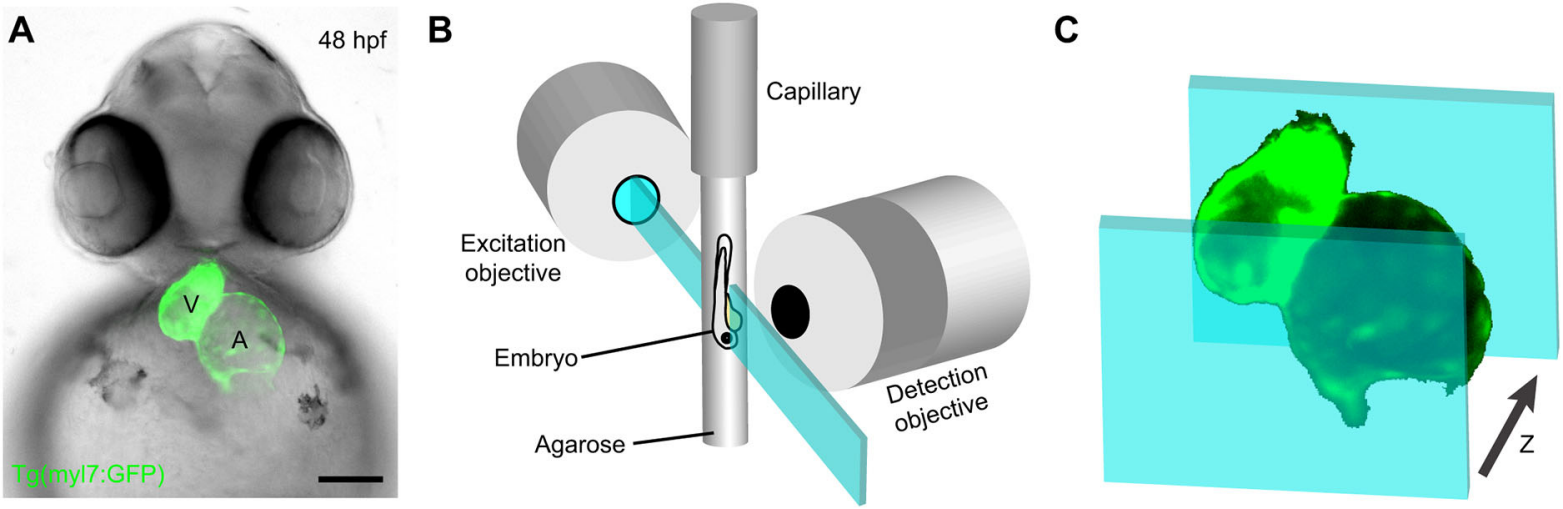
***In vivo* cardiac imaging with light-sheet fluorescence** **microscopy (LSFM).** (A) Fluorescent image of a 48 hpf *Tg(myl7:GFP)* zebrafish embryo with bright-field overlay. Scale bar: 100 μm. (B) Schematic depicting the imaging of a live zebrafish embryo immobilized in agarose within the excitation and detection axis of a light-sheet microscope. (C) Schematic of dynamic image acquisition through *z*-depths.

To image the beating hearts of embryonic zebrafish, we used LSFM to acquire serial dynamic images of 48- to 52-hpf transgenic animals expressing the myocardial-specific *Tg(myl7:GFP)* fluorescent reporter [formerly *Tg(cmlc2:GFP)*] (Burns et al., 2005) (Fig. 1A). Embryos were immobilized in a column of low-melt agarose and submerged in embryo media (EM) within the temperature-controlled incubation chamber of a Zeiss Lightsheet Z.1 microscope (Fig. 1B). We captured dynamic images spanning at least four cardiac cycles at 25.6 frames per second (fps) in 1 μm *z*-depth intervals until the entire heart had been imaged (Fig. 1C). These imaging parameters produced approximately 4500 frames per heart.

**Fig. 2.**
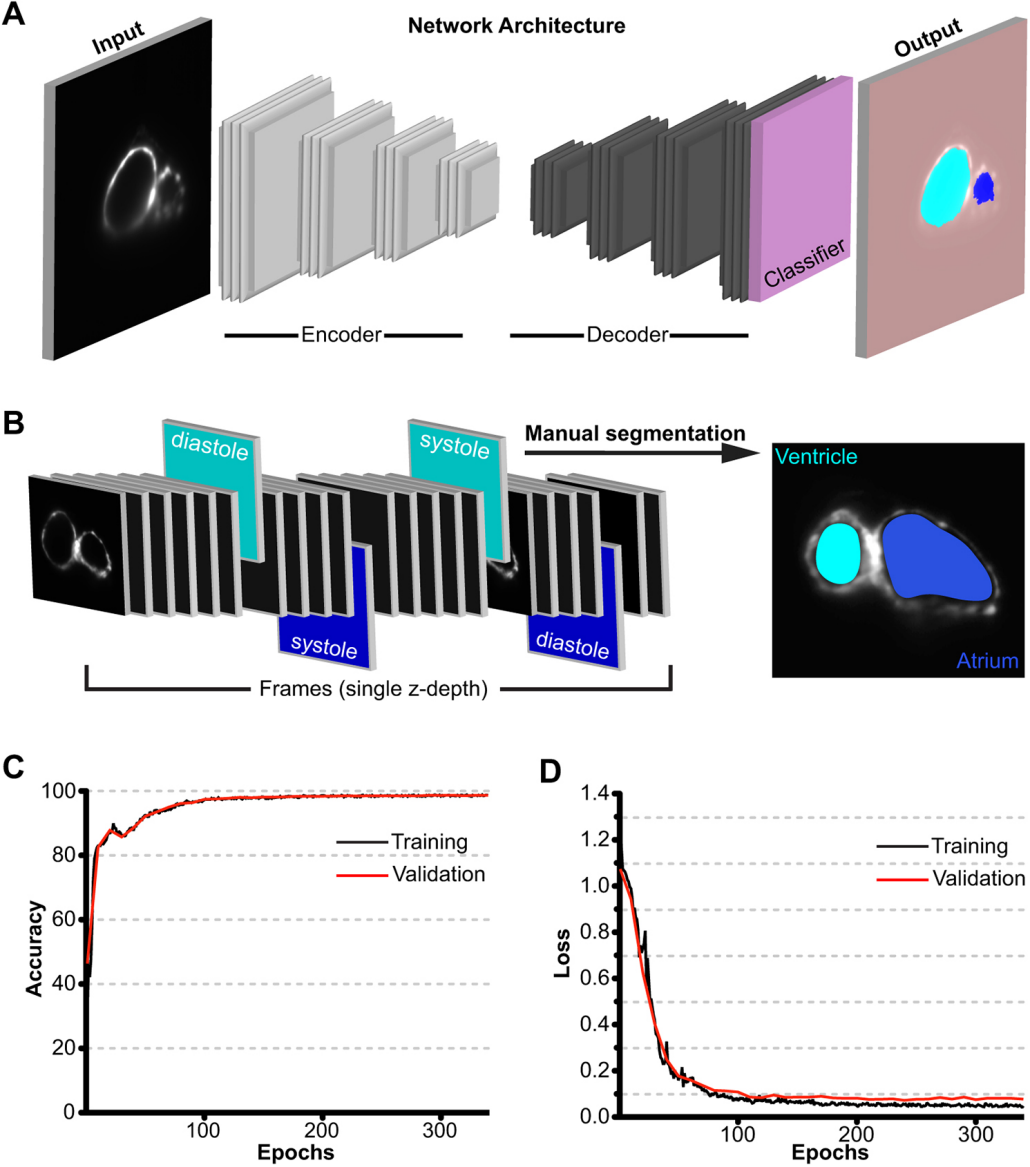
**Neural network design and training.** (A) Conceptual depiction of CFIN's image segmentation workflow: using the SegNet architecture, LSFM images are deconstructed by the encoder to extract local features at varying resolutions, which are then upsampled by the decoder to map each feature weight back to their correct positions relative to the input image. Then, the final decoder layer data is fed to a multi-class Softmax classifier, which assigns each pixel to their respective label of atrium, ventricle or background. (B) Illustration representing the generation of training data via manual segmentation of systole and diastole for each chamber within a dynamic image. Cyan frames and labels indicate ventricle; blue frames and labels indicate atrium. (C) CFIN training and validation accuracy. (D) CFIN training and validation loss. Each epoch represents 30 iterations.

To efficiently process the vast amount of data generated by LSFM, we utilized a deep learning convolutional neural network to automatically identify and annotate the atrial and/or ventricular boundaries in each frame of 2D LSFM movies. This aspect of CFIN was built in MATLAB using the underlying architecture of the deep convolutional encoder-decoder SegNet (Badrinarayanan et al., 2017) (Fig. 2A). To train the network to distinguish atrium, ventricle and background in each frame, we created a ground-truth reference dataset by manually segmenting systole and diastole for both chambers, at each *z*-depth, across four animals (Fig. 2B). This manually annotated dataset amounted to 13% of the total images collected for each heart (≈2400 images total from four hearts). A cross-validation dataset was composed of 230 images from a fifth manually segmented heart, to which the network was naïve. Training was conducted at a variable learning rate beginning at 0.1, which was reduced by a factor of five every two epochs. Using these parameters, we observed an increase in accuracy and a decrease in the loss function for both the training and cross-validation datasets (Fig. 2C,D), which were consistent with successful training. We terminated training when the cross-validation loss function was minimized (Fig. 2D).

**Fig. 3.**
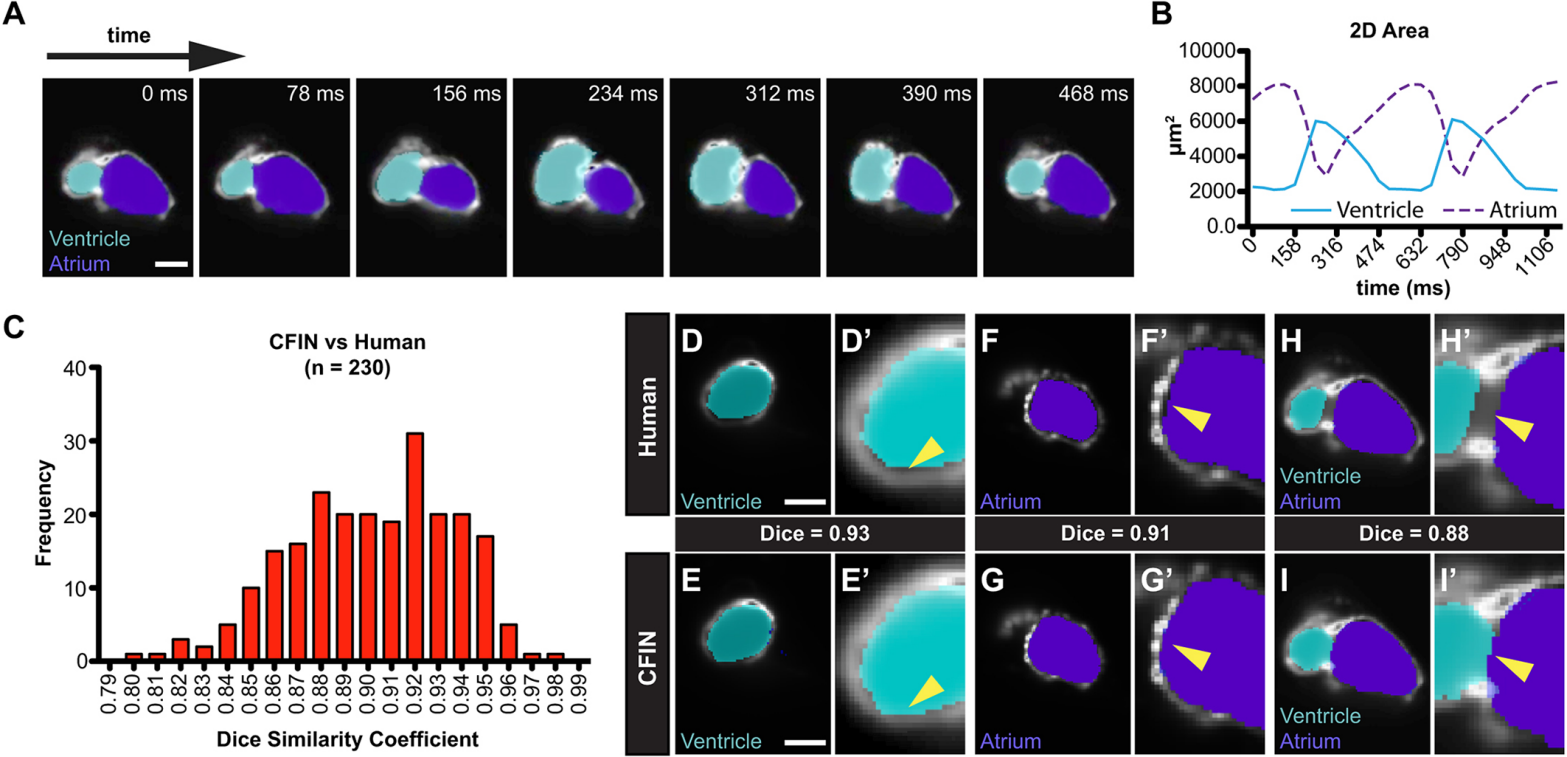
**Validation of CFIN image segmentation.** (A) Sequential frames from a representative dynamic image annotated by CFIN. Ventricle is shown in cyan and atrium in purple. Scale bar: 50 μm. (B) CFIN's quantification of chamber areas from a single dynamic image over time. Atrium (purple dashed); ventricle (blue). (C) Dice similarity coefficient values for 230 validation images. (D-I) Single-frame comparisons of CFIN and human chamber segmentation from *z*-depths containing the ventricle (D,E), the atrium (F,G), or both the atrium and ventricle (H,I). Scale bars: 50 μm.

Following training, we visually assessed the network's ability to correctly segment atrial and ventricular chambers in successive frames of the cross-validation dataset. At every *z*-depth inspected (*n*=10 *z*-depths containing 30 frames each), CFIN successfully segmented and identified each chamber throughout the entire cardiac cycle, including intermediate phases between systole and diastole that were not included in the training dataset (Fig. 3A, Fig. S1). Chamber segmentation also appeared consistent across cardiac phases over multiple cardiac cycles (Movie 1). This can be further appreciated by observing CFIN's chamber area measurements over time, which revealed the expected mirror-image contraction and relaxation patterns of the atrium and ventricle (Fig. 3B). Notably, CFIN's ability to segment both cardiac chambers far surpassed those achieved when non-machine learning-based algorithms were applied to images of fluorescent zebrafish hearts at the same developmental stage (Krämer et al., 2009).

To gauge CFIN's segmentation accuracy within each frame, we generated Dice similarity coefficients comparing its labels to those that were manually annotated for 230 frames within the cross-validation dataset (Zou et al., 2004). Across all images, the average Dice score was 0.9 with a standard deviation (s.d.) of 0.03 (Fig. 3C), demonstrating a high degree of similarity between CFIN and ground-truth labels. In fact, visual comparisons at multiple *z*-depths revealed instances where CFIN's chamber labels were more closely associated with the chamber wall than those that were segmented manually (Fig. 3D-G). Surprisingly, we also found that our network appropriately assigned mutually exclusive regions within the atrioventricular canal to either ventricle or atrium even though this region was deliberately avoided during manual segmentation because absolute boundaries between chambers in this region do not exist (Fig. 3H,I). This demonstrates CFIN's remarkable ability to correctly infer chamber boundaries with a high degree of accuracy while simultaneously overcoming the inherent limitations of our human-computer interface.

To extract volumetric parameters of cardiac function after image segmentation, CFIN identifies the maximal and minimal chamber areas at each *z*-depth. These numbers are multiplied by the *z*-depth interval (1 μm) and summed within each category [maximal (diastole) or minimal (systole); atrium or ventricle] to calculate chamber-specific end diastolic volumes (EDVs) and end systolic volumes (ESVs). From these values we calculate EF, the percentage of blood expelled per beat, for the atrium and ventricle, defined by EF=(EDV−ESV)/EDV. Heart rate (HR) is determined from the periodicity of area measurements and used to calculate CO for each chamber according to CO=HR×(EDV−ESV). In the human population, the left-ventricular EF and CO are the most commonly monitored clinical parameters of heart function (Zipes et al., 2018). In most cases, measuring these parameters for the single zebrafish ventricle would be the most accurate measurement of cardiac performance. However, zebrafish embryos can survive without ventricular function (Auman et al., 2007), making atrial measurements more appropriate in special cases.

**Fig. 4.**
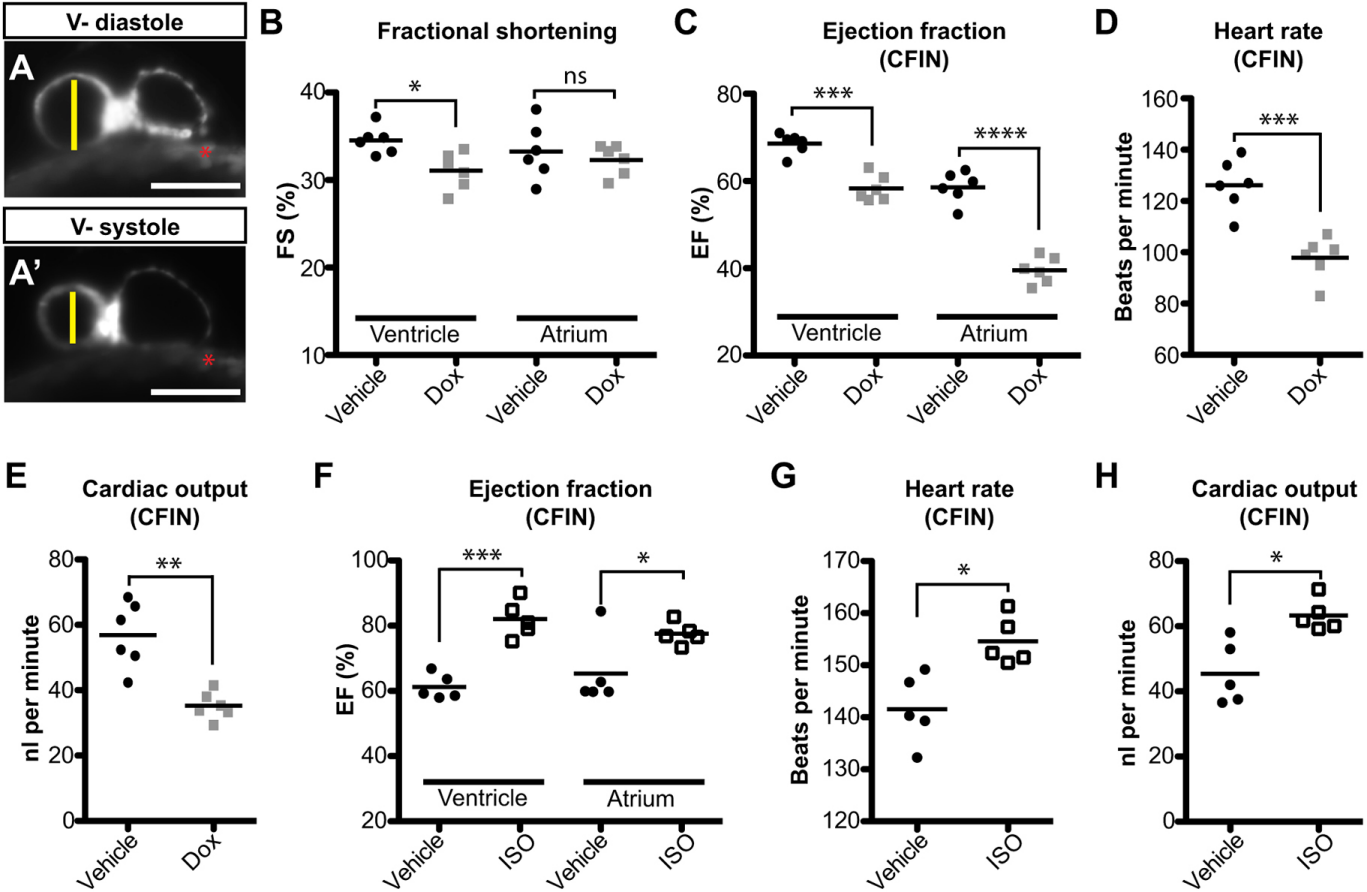
**Experimental validation of CFIN functional assessments of the heart.** (A) Methodology for determining fractional shortening (FS) using EDD (vertical line in A) and ESD (vertical line in A′). Scale bars: 100 μm. Red asterisk denotes Dox autofluorescence. V-, ventricle. (B) FS measurements of doxorubicin (DOX)-treated embryos and control siblings. (C-E) CFIN’s assessment of ejection fraction (EF; C), heart rate (HR; D) and cardiac output (CO; E) within the same experimental cohort. (F-H) CFIN's assessment of EF (F), HR (G) and CO (H) in 48 hpf embryos treated with isoproterenol. Significance is denoted as *P<0.05, **P<0.005, ***P<0.0005, ****P<0.00005, or ns (not significant) as determined by the Student's two-tailed *t*-test.

Next, we tested the hypothesis that volumetric assessments of cardiac function by CFIN could be more sensitive than those based on FS. A previous study demonstrated that zebrafish embryos exposed to doxorubicin (Dox) beginning at 24 hpf exhibit reductions in FS at 72 hpf (Liu et al., 2014). We repeated this experiment but analyzed embryos at 48 hpf, when the functional deficits are likely to be milder. Embryos were imaged using LSFM as described and cardiac function was assessed using FS (Fig. 4A) followed by volumetric assessment by CFIN. Although the FS index detected a small reduction in ventricular function in Dox-treated animals, it was unable to resolve any significant differences in atrial contraction between experimental groups (Fig. 4B). Similarly, calculating EF using estimates of chamber volume, derived from measurements of chamber radii and the assumption that the chambers are spherical, produced similar results (Fig. S2A). Conversely, the EF for both chambers, HR and the CO as measured by CFIN were all clearly reduced following Dox exposure, and presented a much greater statistical significance than FS (Fig. 4C-E). Furthermore, CFIN's performance was unaffected by the presence of Dox-induced autofluorescence in the yolk (Fig. 4A, Fig. S2B,C). These data demonstrate that volumetric measurements made by CFIN can identify functional deficits that may otherwise go unnoticed using FS.

Next, we determined whether CFIN could detect increases in CO. Previous reports have demonstrated that embryonic zebrafish exposed to the β-adrenergic agonist isoproterenol exhibit elevated FS. However, this effect was only detectable when animals 72 hpf or older were exposed to the drug and was not apparent in 48 hpf animals (Kossack et al., 2017; Steele et al., 2011). To determine whether our platform could again detect functional changes that traditional methods were unable to resolve, we treated 48 hpf embryos with isoproterenol for 2 h prior to LSFM imaging. Image analysis with CFIN revealed significant elevations in EF, HR and CO when compared to control animals (Fig. 4F-H). Collectively, these data demonstrate that CFIN is capable of detecting elevations in CO and provides another example of increased sensitivity over FS.

Importantly, the time it takes for CFIN to segment every frame and derive functional parameters from dynamic LSFM images is approximately 3-4 min per heart. This is compared to the ∼2 h required for an expert to manually segment only systole and diastole for each chamber and *z*-depth. Removing the human component in this fashion also offers absolute consistency and removes the potential for errors during manual segmentation, such as poorly drawn labels and incorrectly identified cardiac phases. Because imaging the hearts with LSFM remains the rate-limiting step of our platform, we next explored whether increasing the *z*-depth step size, thus decreasing the time required for imaging, would affect CFINs measurements in experimental conditions. Using our Dox experiment as a model, our data displayed the expected inverse correlation between the step size and accuracy of EF measurements (Fig. S3A,B). Despite the decrease in statistical power that accompanies larger step sizes, CFIN was still able to detect significant differences within each chamber using either a 2 μm or 4 μm step size. These data further demonstrate the robustness of the CFIN platform and allow for the optimization of imaging protocols based on desired functional resolution.

**Fig. 5.**
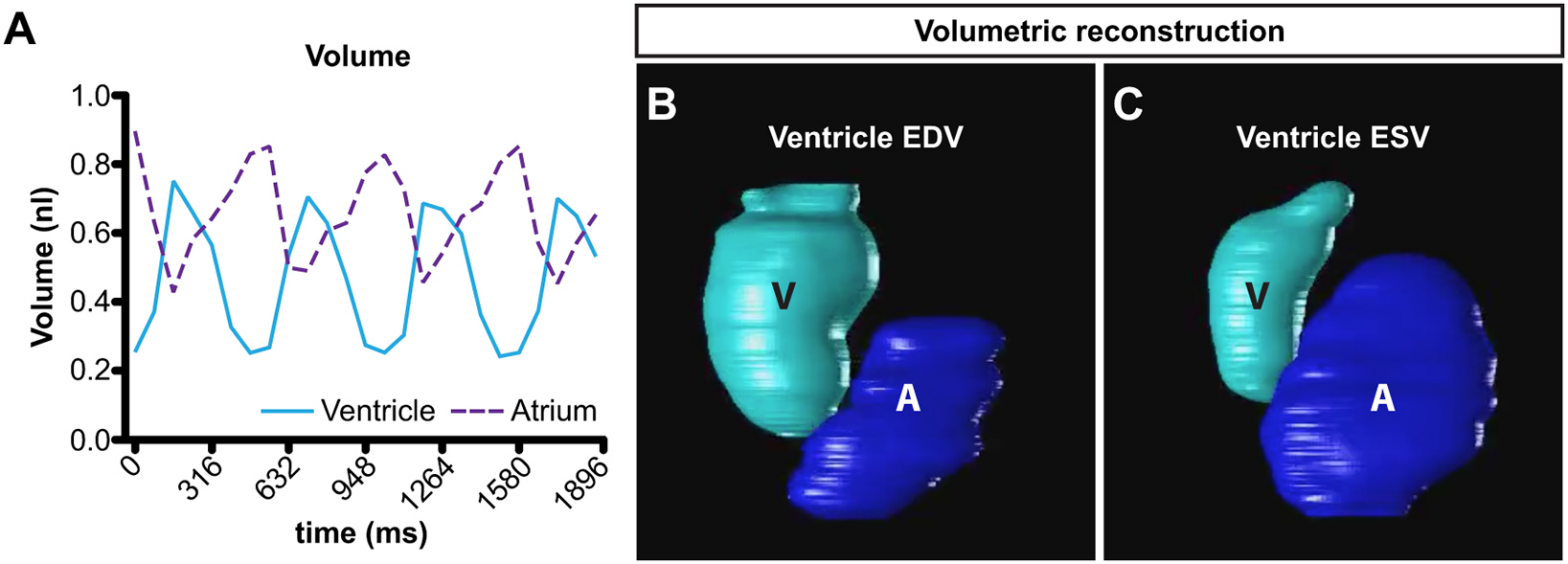
**Volumetric reconstructions using CFIN.** (A) Continuous chamber volumes over three cardiac cycles in a wild-type 48 hpf zebrafish embryo. Volumes were calculated using synchronized images processed by CFIN. Ventricle (cyan); atrium (purple dashed). (B,C) Graphical 3D reconstruction of ventricular EDV (B) and ESV (C) as measured by CFIN. Ventricle (V); atrium (A).

Although CFIN does not require image synchronization or 4D reconstruction to calculate indices of cardiac function such as EF and CO, segmented images can be efficiently used for these purposes, therefore expanding the number of downstream applications. To accomplish this, we synchronized optical sections to the end-diastolic filling period of the atrium as defined by CFIN's measurements of area. In cases where slices did not contain the atrium, we instead used the ventricular period closely corresponding to the atrial end-diastolic filling period identified in the slices with both chambers. Once synchronized in this fashion, images can then be used to observe continuous chamber volume over time (Fig. 5A), which has the potential to reveal complex functional deficits and/or arrhythmias in one or both chambers. Further, graphical 3D and 4D reconstructions built from synchronized data (Fig. 5B,C, Movie 2) have the potential to reveal regional abnormalities in wall movement that may otherwise go unnoticed.

## DISCUSSION

Our platform represents a novel, rapid and robust method for making volumetric assessments of cardiac function in embryonic zebrafish. CFIN greatly increases experimental rigor by not relying on approximations or the inherent assumptions of traditional methods, thereby providing a higher standard of accuracy and consistency. It should be noted that CFIN was specifically developed and validated for use with embryos of approximately 48-55 hpf. Preliminary attempts to apply the platform, as currently configured, to later-stage embryos (72 hpf) resulted in suboptimal chamber segmentation, presumably because the gross morphology of the heart is sufficiently different, and this stage was not included in the training dataset. Nonetheless, later-stage analysis will likely be feasible with additional stage-specific training (i.e. transfer learning).

Given the recent commercialization and increased popularity of light-sheet fluorescence microscopes, we anticipate that our method, as presented here, will be widely accessible to other researchers. Contemporary advances in spinning-disk and resonant scanning confocal microscopes suggest that these imaging modalities will be compatible with CFIN, provided that the image quality and frame rate are sufficiently similar to those parameters in our training dataset. Importantly, our network does not require expensive software, hardware or cloud-based subscriptions and is operated in its entirety via the widely used MATLAB computing environment. Collectively, these attributes make CFIN an accessible and powerful new tool to accurately measure heart function in an influential vertebrate model organism, which will ultimately advance our understanding of heart failure.

## MATERIALS AND METHODS

### Zebrafish

Zebrafish were bred and maintained following protocols approved by the Massachusetts General Hospital Institutional Animal Care and Use Committee (IACUC), and adhere to the recommendations outlined by the Guide for the Care and Use of Laboratory Animals (National Academic Press). The following zebrafish (*Danio rerio*) strain was used in this study: *Tg(myl7:GFP)* (Burns et al., 2005).

### Microscopy

For compound microscopy, zebrafish embryos were anesthetized in 0.16% tricaine (Western Chemical) and immobilized in 0.9% low-melt agarose dissolved in EM. Embryos were harvested at stages ≥48 hpf and imaged on depression slides using epifluorescence or bright-field optics on a Nikon Eclipse 80i compound microscope.

For light-sheet microscopy, embryos were treated with 0.003% phenylthiourea (PTU) at 24 hpf to prevent pigmentation. At 48 hpf, treated embryos were briefly anesthetized and immobilized in 0.9% low-melt agarose within a glass capillary (Zeiss). The capillary was then submerged in temperature-controlled EM with 0.016% tricaine and imaged with the ZEISS Lightsheet Z.1 microscope. Live-mounted *Tg(myl7:GFP)* zebrafish were imaged through the entire ventricle by collecting dynamic plane images at each GPF-positive *z*-depth. Dynamic images were taken every 1 μm and spanned at least four cycles of the beating heart. Images were taken at each *z*-depth until the entire heart had been recorded.

### Training the network

Training datasets were generated from five animals across two separate crosses that were imaged on different days. Image brightness was normalized prior to training and/or analysis. To minimize the potential training bias caused by a large number of background pixels (primarily coming from yolk autofluorescence), pixel classification weights were set as the reciprocal of the frequency of each classification (‘atrium’=38.2, ‘ventricle’=64.9, ‘background’=1). CFIN utilizes the underlying architecture of SegNet, with an encoder depth of 4 and an input image matrix of 160×160 pixels to reduce the time required for training and analysis. Training data was created via manual segmentation of each chamber in 2400 static images using MATLAB.

### Synchronization and volumetric reconstruction

After running CFIN to initially annotate each 2D image of the entire 4D dataset, we performed synchronization by first identifying the end-diastolic atrial cardiac phase, *t*_EDA, that maximizes the cost parameter, phi(*t*), for each 2D temporal image at each *z*-stack:
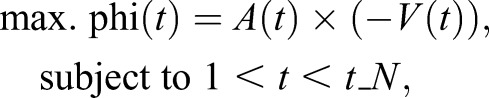
where *A*(*t*), *V*(*t*) and *t*_*N* represent the area of labeled atrium pixels, area of labeled ventricle pixels and maximum number of temporal images acquired, respectively. Once the *t*_EDA is determined for each *z*-stack, we re-index the 2D temporal images at each *z*-stack to start at the *t*_EDA. Then, one period of the cardiac cycle is identified by searching forward for the next *t*_EDA maximizing again phi(*t*) for each *z*-stack. Finally, the single period of cardiac cycle is temporally cropped and interpolated to 24.6 fps. This ensures that any slight arrhythmias that may occur between *z*-stacks are accounted. At each cardiac time frame, 3D reconstruction surface models were constructed using the segmented atrial and ventricular binary masks generated from CFIN. Then, the 3D reconstructions were played dynamically at the acquired temporal resolution of the LSFM.

### Doxorubicin treatment

Stock Dox (Sigma Aldrich) was reconstituted in Milli-Q water and stored at 4°C in the dark. At 24 hpf, embryos from the same clutch were randomly selected to be treated with 100 μM Dox or an equivalent volume of Milli-Q (vehicle) diluted in EM. Embryos were kept in their respective solution until imaging at 48 hpf, prior to which they were briefly rinsed in EM to reduce autofluorescence caused by excess Dox. Data were collected over two separate experiments performed on different days and with different animals.

### Isoproterenol treatment

Isoproterenol hydrochloride (Sigma Aldrich) was reconstituted in EM fresh before each use. At 48 hpf, embryos were randomly selected within each clutch to be treated with 20 μM isoproterenol in EM for 2 h prior to imaging. Embryos were then anesthetized and imbedded in agarose as described above, and re-submerged in EM containing 20 μM isoproterenol for the duration of imaging. Sibling controls were processed and imaged as described above. Data were collected over two separate experiments performed on different days and with different animals.

### Functional analysis

FS was measured in ImageJ using end-diastolic dimension (EDD) and end-systolic dimension (ESD) of LSFM optical sections. FS was then defined as (EDD–ESD)/EDD×100. Optical sections through the middle of each chamber were chosen for measurements. Chamber diameters were measured within each frame of the optical sections to ensure an accurate description of both diastole and systole. Estimated EF was calculated similarly to previously described methods (Shin et al., 2010), which were modified to assume a spherical, rather than prolate spheroid, chamber morphology because the relative angle of the light sheet precludes measurement of the long axis at a single *z*-depth. FS and EF/CO measurements were derived using the same datasets in order to eliminate variability between cohorts. Each point within Fig. 4B-H represents data from distinct hearts within its respective analysis. The data presented in Fig. S3 represents the same Dox experiment with increased step size simulated through data subtraction. Significance in all functional analyses was determined using the Student's two-tailed *t*-test.

## Supplementary Material

Supplementary information

## References

[DMM040188C1] Auman H. J., Coleman H., Riley H. E., Olale F., Tsai H.-J. and Yelon D. (2007). Functional modulation of cardiac form through regionally confined cell shape changes. *PLoS Biol.* 5, e53 10.1371/journal.pbio.0050053 17311471 PMC1802756

[DMM040188C2] Badrinarayanan V., Kendall A. and Cipolla R. (2017). SegNet: a deep convolutional encoder-decoder architecture for image segmentation. *IEEE Trans. Pattern Anal. Mach. Intell.* 39, 2481-2495. 10.1109/TPAMI.2016.2644615 28060704

[DMM040188C3] Bai W., Sinclair M., Tarroni G., Oktay O., Rajchl M., Vaillant G., Lee A. M., Aung N., Lukaschuk E., Sanghvi M. M. et al. (2018). Automated cardiovascular magnetic resonance image analysis with fully convolutional networks. *J. Cardiovasc. Magn. Reson.* 20, 65 10.1186/s12968-018-0471-x 30217194 PMC6138894

[DMM040188C4] Bakkers J. (2011). Zebrafish as a model to study cardiac development and human cardiac disease. *Cardiovasc. Res.* 91, 279-288. 10.1093/cvr/cvr098 21602174 PMC3125074

[DMM040188C5] Becker J. R., Robinson T. Y., Sachidanandan C., Kelly A. E., Coy S., Peterson R. T. and MacRae C. A. (2012). In vivo natriuretic peptide reporter assay identifies chemical modifiers of hypertrophic cardiomyopathy signalling. *Cardiovasc. Res.* 93, 463-470. 10.1093/cvr/cvr350 22198505 PMC3410427

[DMM040188C6] Burns C. G., Milan D. J., Grande E. J., Rottbauer W., MacRae C. A. and Fishman M. C. (2005). High-throughput assay for small molecules that modulate zebrafish embryonic heart rate. *Nat. Chem. Biol.* 1, 263-264. 10.1038/nchembio732 16408054

[DMM040188C7] Chen J. N., Haffter P., Odenthal J., Vogelsang E., Brand M., van Eeden F. J., Furutani-Seiki M., Granato M., Hammerschmidt M., Heisenberg C. P. et al. (1996). Mutations affecting the cardiovascular system and other internal organs in zebrafish. *Development* 123, 293-302. 10.1007/s004270050051 9007249

[DMM040188C8] Ciompi F., de Hoop B., van Riel S. J., Chung K., Scholten E. T., Oudkerk M., de Jong P. A., Prokop M. and van Ginneken B. (2015). Automatic classification of pulmonary peri-fissural nodules in computed tomography using an ensemble of 2D views and a convolutional neural network out-of-the-box. *Med. Image Anal.* 26, 195-202. 10.1016/j.media.2015.08.001 26458112

[DMM040188C9] Dou Q., Yu L., Chen H., Jin Y., Yang X., Qin J. and Heng P.-A. (2017). 3D deeply supervised network for automated segmentation of volumetric medical images. *Med. Image Anal.* 41, 40-54. 10.1016/j.media.2017.05.001 28526212

[DMM040188C10] Fei P., Lee J., Packard R. R. S., Sereti K.-I., Xu H., Ma J., Ding Y., Kang H., Chen H., Sung K. et al. (2016). Cardiac light-sheet fluorescent microscopy for multi-scale and rapid imaging of architecture and function. *Sci. Rep.* 6, 22489 10.1038/srep22489 26935567 PMC4776137

[DMM040188C11] Hay E. A. and Parthasarathy R. (2018). Performance of convolutional neural networks for identification of bacteria in 3D microscopy datasets. *PLoS Comput. Biol.* 14, e1006628 10.1371/journal.pcbi.1006628 30507940 PMC6292638

[DMM040188C12] Hoage T., Ding Y. and Xu X. (2012). Quantifying cardiac functions in embryonic and adult zebrafish. *Methods Mol. Biol.* 843, 11-20. 10.1007/978-1-61779-523-7_2 22222517 PMC3762588

[DMM040188C13] Kossack M., Hein S., Juergensen L., Siragusa M., Benz A., Katus H. A., Most P. and Hassel D. (2017). Induction of cardiac dysfunction in developing and adult zebrafish by chronic isoproterenol stimulation. *J. Mol. Cell Cardiol.* 108, 95-105. 10.1016/j.yjmcc.2017.05.011 28554511

[DMM040188C14] Krämer P., Boto F., Wald D., Bessy F., Paloc C., Callol C., Letamendia A., Ibarbia I., Holgado O. and Virto J. M. (2009). Comparison of segmentation algorithms for the zebrafish heart in fluorescent microscopy images. In *Advances in Visual Computing*, (ed. G. Bebis, R. Boyle, B. Parvin, D. Koracin, Y. Kuno, J. Wang, R. Pajarola, P. Lindstrom, A. Hinkenjann, M. L. Encarnacao, C. T. Silva and D. Coming), pp. 1041-1050. Springer, Berlin, Heidelberg.

[DMM040188C15] Lee J., Fei P., Packard R. R. S., Kang H., Xu H., Baek K. I., Jen N., Chen J., Yen H., Kuo C.-C. J. et al. (2016). 4-Dimensional light-sheet microscopy to elucidate shear stress modulation of cardiac trabeculation. *J. Clin. Invest.* 126, 1679-1690. 10.1172/JCI83496 27018592 PMC4855946

[DMM040188C16] Litjens G., Kooi T., Bejnordi B. E., Setio A. A. A., Ciompi F., Ghafoorian M., van der Laak J. A. W. M., van Ginneken B. and Sánchez C. I. (2017). A survey on deep learning in medical image analysis. *Med. Image Anal.* 42, 60-88. 10.1016/j.media.2017.07.005 28778026

[DMM040188C17] Liu Y., Asnani A., Zou L., Bentley V. L., Yu M., Wang Y., Dellaire G., Sarkar K. S., Dai M., Chen H. H. et al. (2014). Visnagin protects against doxorubicin-induced cardiomyopathy through modulation of mitochondrial malate dehydrogenase. *Sci. Transl. Med.* 6, 266ra170 10.1126/scitranslmed.3010189 PMC4360984 25504881

[DMM040188C18] Mickoleit M., Schmid B., Weber M., Fahrbach F. O., Hombach S., Reischauer S. and Huisken J. (2014). High-resolution reconstruction of the beating zebrafish heart. *Nat. Methods* 11, 919-922. 10.1038/nmeth.3037 25042787

[DMM040188C19] Packard R. R. S., Baek K. I., Beebe T., Jen N., Ding Y., Shi F., Fei P., Kang B. J., Chen P.-H., Gau J. et al. (2017). Automated segmentation of light-sheet fluorescent imaging to characterize experimental doxorubicin-induced cardiac injury and repair. *Sci. Rep.* 7, 8603 10.1038/s41598-017-09152-x 28819303 PMC5561066

[DMM040188C20] Sharma N. and Aggarwal L. M. (2010). Automated medical image segmentation techniques. *J. Med. Phys.* 35, 3-14. 10.4103/0971-6203.58777 20177565 PMC2825001

[DMM040188C21] Shih Y.-H., Zhang Y., Ding Y., Ross C. A., Li H., Olson T. M. and Xu X. (2015). Cardiac transcriptome and dilated cardiomyopathy genes in zebrafish. *Circ. Cardiovasc. Genet.* 8, 261-269. 10.1161/CIRCGENETICS.114.000702 25583992 PMC4406804

[DMM040188C22] Shin J. T., Pomerantsev E. V., Mably J. D. and MacRae C. A. (2010). High-resolution cardiovascular function confirms functional orthology of myocardial contractility pathways in zebrafish. *Physiol. Genomics* 42, 300-309. 10.1152/physiolgenomics.00206.2009 20388839 PMC3032279

[DMM040188C23] Stainier D. Y., Fouquet B., Chen J. N., Warren K. S., Weinstein B. M., Meiler S. E., Mohideen M. A., Neuhauss S. C., Solnica-Krezel L., Schier A. F. et al. (1996). Mutations affecting the formation and function of the cardiovascular system in the zebrafish embryo. *Development* 123, 285-292. 9007248 10.1242/dev.123.1.285

[DMM040188C24] Steele S. L., Yang X., Debiais-Thibaud M., Schwerte T., Pelster B., Ekker M., Tiberi M. and Perry S. F. (2011). In vivo and in vitro assessment of cardiac beta-adrenergic receptors in larval zebrafish (Danio rerio). *J. Exp. Biol.* 214, 1445-1457. 10.1242/jeb.052803 21490253

[DMM040188C25] Yalcin H. C., Amindari A., Butcher J. T., Althani A. and Yacoub M. (2017). Heart function and hemodynamic analysis for zebrafish embryos. *Dev. Dyn.* 246, 868-880. 10.1002/dvdy.24497 28249360

[DMM040188C26] Zhang Y., Chen J.-H., Chang K.-T., Park V. Y., Kim M. J., Chan S., Chang P., Chow D., Luk A., Kwong T. et al. (2019). Automatic breast and fibroglandular tissue segmentation in breast MRI using deep learning by a fully-convolutional residual neural network U-net. *Acad. Radiol*. (in press). 10.1016/j.acra.2019.01.012 PMC6669125 30713130

[DMM040188C27] Zipes D. P., Libby P., Bonow R. O., Mann D. L. and Tomaselli G. F. (2018). *Braunwald's Heart Disease E-Book*. Elsevier Health Sciences.

[DMM040188C28] Zou K. H., Warfield S. K., Bharatha A., Tempany C. M. C., Kaus M. R., Haker S. J., Wells W. M. III, Jolesz F. A. and Kikinis R. (2004). Statistical validation of image segmentation quality based on a spatial overlap index. *Acad. Radiol.* 11, 178-189. 10.1016/S1076-6332(03)00671-8 14974593 PMC1415224

