# Supplementary material for: Deep learning enables automated volumetric assessments of cardiac function in zebrafish
Source: Dis Model Mech. 2019 Oct 25;12(10):dmm040188. doi: 10.1242/dmm.040188 (PMC6826023; doi:10.1242/dmm.040188)
Supplement: Supplementary information [file dmm-12-040188-s1.pdf]

SUPPLEMENTARY INFORMATION

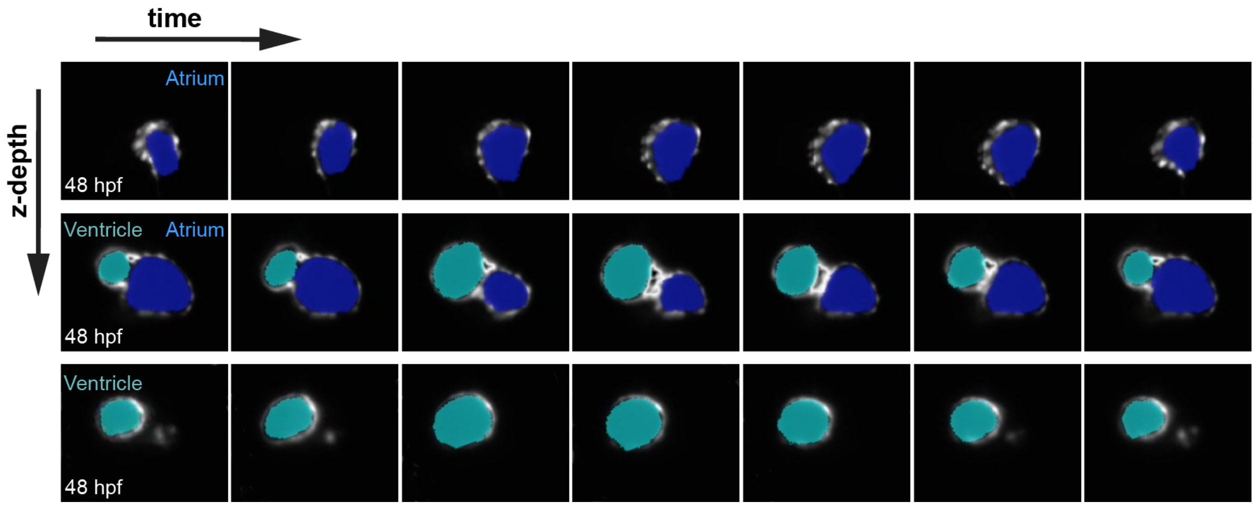

Figure S1: CFIN chamber classifications at distinct z-depths.
CFIN-generated chamber labels over a single cardiac cycle at three representative z-depths displaying only atrium, atrium and ventricle, or only ventricle.

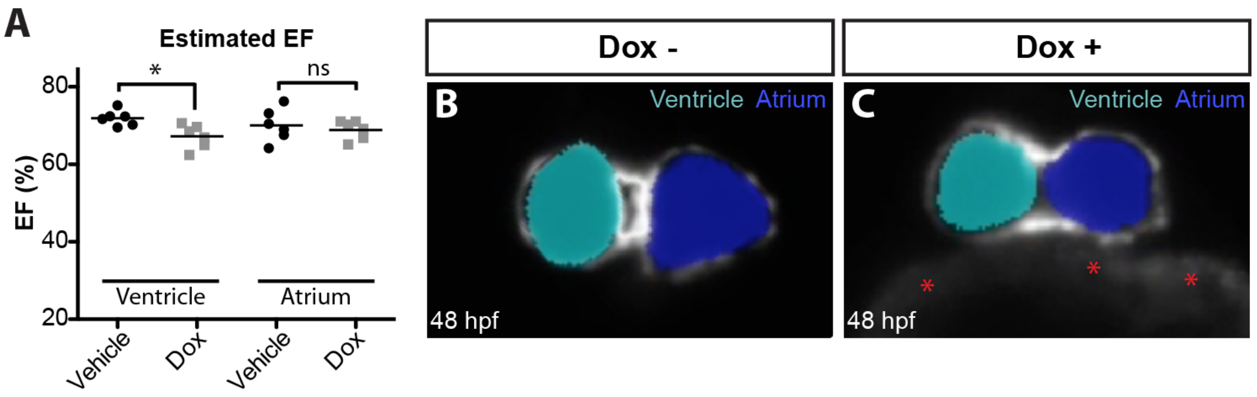

Figure S2: Estimated EF and Dox-induced autofluorescence.
(**A**) Estimated ejection fraction values of Dox treated embryos and controls using chamber radius measurements and the assumption of spherical chambers. (**B-C**) CFIN labels in untreated embryos (B) compared with Dox treated (C) in the presence of Dox autofluorescence in the yolk (red asterisks).

Disease Models & Mechanisms • Supplementary information

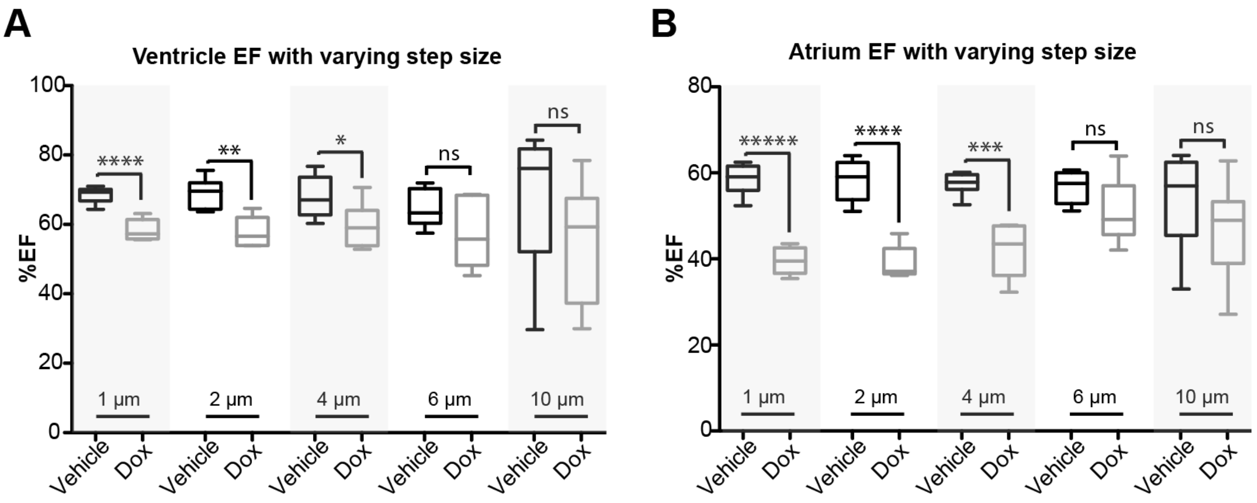

Figure S3: The effects of increased step size on volume resolution. (**A-B**) Ventricular (A) and Atrial (B) EF measurements produced by CFIN using input datasets with increasing z-depth step sizes. Significance denoted as *****$p < 10^{-5}$, ****$p < 10^{-4}$, ***$p < 10^{-3}$, **$p < 10^{-2}$, *$p < 0.01$, or ns (not significant $p > 0.05$) as determined by the students two tailed t-test.

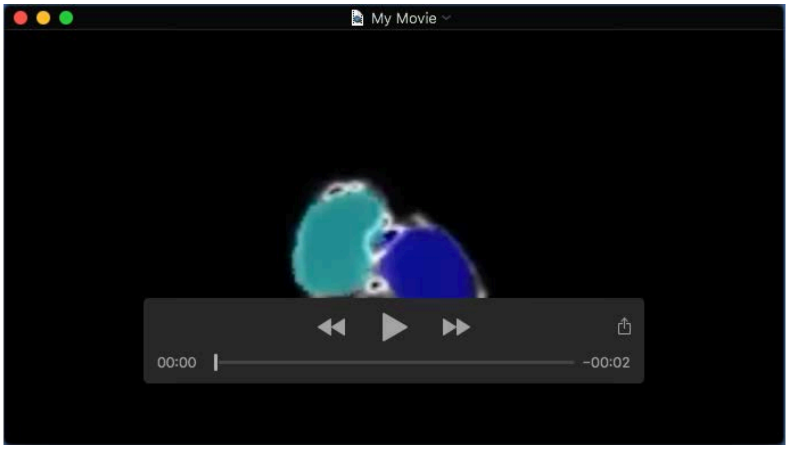

Movie 1: CFIN validation at a single z-depth

Dynamic image from a single z-depth with CFIN chamber annotations overlaid.

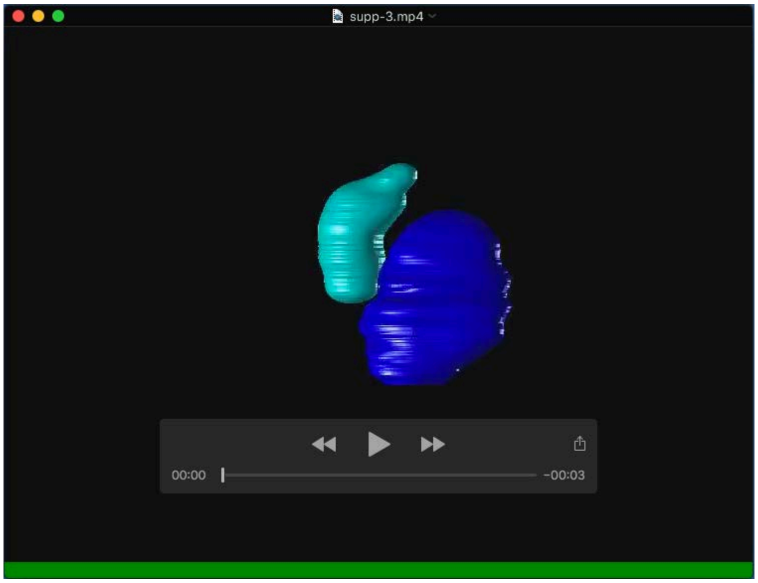

Movie 2: Volumetric 4D reconstruction from CFIN analysis

4D graphical reconstruction generated with a synchronized dataset analyzed by CFIN.

Disease Models & Mechanisms • Supplementary information

CFIN_final.mat
This file contains the trained network for image segmentation of embryonic zebrafish hearts. (To be opened in MATLAB.)

CFIN_Code.m
This file contains the MATLAB code for volumetric analysis of segmented images.
